# Predictive factors of prolonged mechanical ventilation, overall survival, and quality of life in patients with post-thymectomy myasthenic crisis

**DOI:** 10.1186/s12957-017-1209-1

**Published:** 2017-08-08

**Authors:** Kun-Kun Li, Kai Qian, Yong-Geng Feng, Wei Guo, Qun-You Tan, Bo Deng

**Affiliations:** Department of Thoracic Surgery, Institute of Surgery Research, Daping Hospital, Third Military Medical University, Chongqing, 400042 People’s Republic of China

**Keywords:** Post-thymectomy, Myasthenic, Crisis·mechanical, Ventilation, Survival, Quality of life

## Abstract

**Background:**

Thymectomy is the primary approach for the treatment of myasthenia gravis (MG). This retrospective study aimed to identify the clinical and demographical features that may impact the duration of mechanical ventilation (DMV), the long-term survival, and the quality of life (QOL) in patients with post-thymectomy myasthenic crisis (PTMC).

**Methods:**

We reviewed the patients who suffered from PTMC from June 2008 to November 2015. Cox proportional hazard regression analysis was used to identify potential prognostic factors that may impact DMV and long-term survival. Spearman bivariate correlation analysis was used to analyze the relationship between DMV and QOL. Statistical powers were calculated.

**Results:**

In total, 70 patients with PTMC were enrolled. Alcohol abuse, high scores of Myasthenia Gravis Foundation of America (MGFA) classification and Clavien-Dindo classification were critical factors that remarkably delayed early extubation. High scores of Osserman’s classification, MGFA classification, and Clavien-Dindo classification predicted a poor prognosis in PTMC patients. Occupational skills and job status were observed to be negatively affected in PTMC patients.

**Conclusions:**

To decrease the duration of mechanical ventilation, we suggest alcohol abstinence before the operation, appropriate preoperative treatment to decrease MGFA classification, and greater attention to the treatment of postoperative complications.

**Electronic supplementary material:**

The online version of this article (doi:10.1186/s12957-017-1209-1) contains supplementary material, which is available to authorized users.

## Background

Myasthenia gravis (MG) is an acquired autoimmune disorder of neuromuscular junction transmission that clinically manifests as fluctuating weakness and fatigability of skeletal muscles that is usually mediated by auto-antibodies, especially against the human nicotinic acetylcholine receptor (AChR) of postsynaptic membranes [1]. Thymus gland abnormality in 70–90% of MG patients was thought to play a critical role in the pathogenesis of MG [2, 3]. Therefore, thymectomy is believed to be a critical treatment regarding overall survival, clinical improvement, and remission rate and should be considered strongly in patients with generalized MG [4].

However, post-thymectomy myasthenic crisis (PTMC) remains a major challenge necessitating postoperative mechanical ventilatory support and potentially leads to postoperative death. It is defined as mechanical ventilation lasting more than 48 h after thymectomy and/or occurring abruptly and requiring urgent intubation or tracheotomy [5, 6].

Several studies unveiled the risk factors of PTMC, i.e., unstable symptoms of MG before surgery, patients with myasthenic crisis history, thymoma, large preoperative dose of pyridostigmine bromide, postoperative pulmonary infection, and preoperative anxiety (Beck score >8) [7–9]. Furthermore, Lu W et al. [10] found that preoperative Myasthenia Gravis Foundation of America (MGFA) clinical classification and quantitative myasthenia gravis (QMG) score were independent risk factors for prolonged postoperative intubation in patients with MG by logistic regression analysis. However, notably few studies have been conducted that concern the risk factors that may prolong duration of mechanical ventilation (DMV) and impact long-term survival and quality of life (QOL) in patients with PTMC.

In this study, we aim to identify the clinical and demographical features that may impact the abovementioned points regarding the progression and prognosis of PTMC.

## Methods

### Patients

From June 2008 to November 2015, a total of 384 patients suffering from MG underwent extended thymectomy in our institution, and 70 patients had PTMC. The data were collected retrospectively by chart review regarding relevant patient data and clinical variables, and we obtained written informed consent prior to the operation. The study was approved by the Ethics Committee of Daping Hospital.

The diagnosis of MG was based on the case history, clinical manifestation, physical examination, and prostigmin test. PTMC is defined as mechanical ventilation lasting more than 48 h after thymectomy and/or occurring abruptly and requiring urgent intubation or tracheotomy in MG patients [5, 6]. DMV is defined as the duration from trachea intubation to extubation.

The clinical criteria for postoperative lung infection (PLI) include the following: (1) new or progressive lung infiltrate and (2) at least two of the following manifestations: hyper- or hypothermia, elevated white blood cell count, purulent tracheal secretions or sputum, or worsening oxygenation [11].

Osserman’s classification [12] is used to determine the clinical status of the patients, separating patients with purely ocular involvement from those with generalized weakness and, further, separating those with mild, moderate, acute fulminating, and late severe generalized weakness.

MGFA classification is another class system [13]; it defers quantitative assessment of muscle weakness to the more precise Quantitative MG Score for disease severity. According to MGFA, MG can be divided into five primary classes: class I is purely ocular weakness and classes II–IV are generalized weakness, and the subclasses are divided by the limb, axial muscles, or oropharyngeal muscles.

The QMG score [14] is a 13-item scale that is used to quantify disease severity in MG. The scale measures ocular, bulbar, respiratory, and limb function; grades each finding; and ranges from 0 (no myasthenic findings) to 39 (maximal myasthenic deficits).

The Clavien-Dindo classification [15] was used to evaluate the postoperative complications, and we redefined grade 4 in this study: (i) grade 0: no complications, (ii) grade 1: deviation from normal postoperative course without need for medical intervention, (iii) grade 2: complications requiring pharmacological treatment, (iv) grade 3: complications requiring invasive intervention, (v) grade 4: life-threatening complications requiring intensive care unit management, *except* trachea intubation, and (vi) grade 5: death.

### Follow-up

After surgery, all patients were followed up regularly in the outpatient clinic or by telephone every 3 months during the first year, every 6 months until the fifth year, and then annually. All included subjects had complete follow-up information until death or February 2016; the dose of prednisolone or pyridostigmine bromide, the QMG score, the myasthenia gravis quality of life questionnaire (MG-QOL15) (a system for the evaluation of the quality of life in MG patients), the occurrence, and the induced causes of myasthenic crisis (MC) were recorded. We used overall survival (OS) as the primary endpoint that was defined as the time from diagnosis to death.

Fifty-six cases have valid follow-up data, and 14 cases were lost in follow-up (14/70, 20%). The duration of follow-up ranged from 1.2 to 92.8 months (41.5 ± 21.2 months). Patients whose follow-up time was less than 30 days or who died within 30 days after surgery were excluded.

### Statistical analysis

Numerical data are expressed as the mean ± standard deviation. Discrete data are expressed as median (range). Univariate and multivariate Cox proportional hazard regression analyses were used to identify potential prognostic factors. One-way ANOVA analysis was used to compare the four groups with continuous variates. Spearman bivariate correlation analysis was used to analyze the relationship between DMV and QOL. Statistical powers of the Cox model and one-way ANOVA analysis were evaluated by using XLSTAT (Addinsoft Inc., New York, NY, USA). All statistical calculations were performed using the SPSS statistical software, version 19.0 (IBM SPSS, Chicago, IL, USA). A *p* value of <0.05 was considered to be statistically significant.

## Results

### Characteristics of patients suffering from PTMC

In total, 70 patients with PTMC were enrolled in this study. PTMC occurred within 1.0 ± 2.6 days after the thymectomy (ranged from 0 to 19 days); the clinical and demographical characteristics of these patients were summarized in Table 1. Sixty-nine (98.6%) patients had medical treatments prior to operation, i.e., prednisolone, pyridostigmine bromide, plasma exchange, steroid pulse, and/or immunoglobulin. Eleven patients (15.7%) had autoimmune diseases, i.e., five with hyperthyroidism, three with hypothyroidism, one with gout, one with rheumatoid arthritis, and one (1.4%) with systemic lupus erythematosus.

**Table 1:** Clinical and demographic characteristics of PTMC patients

Variable	Number (%) or mean (SD)
Age (years)	39.6 (14.8)
Gender	
Male	31 (44.3%)
Female	39 (55.7%)
BMI	22.3 (3.6)
Symptom duration (months)	21.1 (38.7)
Osserman’s classification	
I	5 (7.1%)
IIa	28 (40.0%)
IIb	30 (42.9%)
III	5 (7.1%)
IV	2 (2.9%)
MGFA classification	
I	5 (7.1%)
IIa	13 (18.6%)
IIb	16 (22.9%)
IIIa	9 (12.9%)
IIIb	21 (30.0%)
IVa	1 (1.4%)
IVb	2 (2.9%)
V	3 (4.3%)
QMG score	14.1 (5.7)
Preoperative medical therapy	69 (98.6%)
Prednisolone	25 (21.4%), 8.2 (19.4)
Plasma exchange	4 (5.7%)
Steroid pulse	9 (12.9%)
Immunoglobulin	4 (5.7%)
Pyridostigmine bromide	69 (98.6%)
No preoperative medical therapy	1 (1.4%)
Autoimmune diseases	
Hyperthyroidism	5 (7.1%)
Hypothyroidism	3 (4.3%)
Gout	1 (1.4%)
Rheumatoid arthritis	1 (1.4%)
Systemic lupus erythematosus	1 (1.4%)
Without autoimmune diseases	59 (84.3%)
Operation approach	
VATS	53 (75.7%)
Transsternal	17 (24.3%)
Clavien-Dindo classification^a^	
0	18 (25.7%)
I	1 (1.4%)
II	41 (58.6%)
IIIa	4 (5.7%)
IIIb	2 (2.9%)
IVa	4 (5.7%)
Mechanical ventilation duration (h)	203.0 (436.3)
Duration of hospital stay (days)	30.3 (28.3)

^a^(i) Grade 0: no complications, (ii) grade 1: deviation from normal postoperative course without need for medical intervention, (iii) grade 2: complications requiring pharmacological treatment, (iv) grade 3: complications requiring invasive intervention, (v) grade 4: life-threatening complications requiring intensive care unit management except trachea intubation, and (vi) grade 5: death [11]

There was no perioperative mortality. Eighteen (25.7%) patients did not have any postoperative complication, except PTMC. Fifty-two (74.3%) cases had other surgical complications that were graded by Clavien classification and ranged from 0 to 5 [15].

Among the 70 cases, the median DMV was 120 (24–3696) h. The median hospital stay duration was 26 (8–240) days.

### Treatment protocols for PTMC

Among the 70 cases, 42 patients prolonged mechanical ventilation more than 48 h, and 28 patients developed MC abruptly and required urgent intubation or a tracheotomy in MG patients. The treatment protocols for PTMC in our experiences included the following. First, we evaluated the type of myasthenic crisis according to prostigmin tests. For a positive result, we increased the dosage of pyridostigmine bromide gradually; for a negative result, we stopped the anticholinesterase drugs completely because we were unable to distinguish the MC type, stopped mechanical ventilation assistance, and evaluated the symptoms again after 24 h while increasing the pyridostigmine bromide from a small dosage. Second, if the dosage of pyridostigmine bromide was more than 360 mg/day, it is important to add the prednisolone acetate from a small dosage. Lastly, for patients who needed more than 10 days of mechanical ventilation, we chose steroid pulse therapy, immunoglobulin, or plasma exchange.

### Risk factors of prolonged mechanical ventilation

We chose 16 variables for Cox model analysis as the potential risk factors according to the reported information shown in Additional file 1: Table S1. The variables were classified and clustered as follows: (i) demographical characteristics, i.e., age, body mass index (BMI), smoking status, alcohol status, and lung function; (ii) status of MG prior to thymectomy, i.e., preoperative crisis, symptom duration, Osserman’s classification, MGFA classification, QMG scores, thymoma, and preoperative max pyridostigmine; (iii) surgical situations, i.e., surgical approaches, operation duration, and blood loss; and (iv) postoperative situations, i.e., Clavien-Dindo classification of surgical complications.

We defined “status = 1” as the tracheal extubation and “time” as the DMV. As shown in Table 2, both univariable and multivariable analyses indicated alcohol status, MGFA classification, and Clavien-Dindo classification were critical factors that may hinder early extubation.

**Table 2:** Univariate and multivariate analyses of risk factors that may prolong mechanical ventilation

Variables	Cox model	*p* value	Hazard ratio (95.0% CI for HR)
Alcohol status^b^	Univariate analysis	0.040	0.524 (0.283–0.970)
	Multivariate analysis^a^	0.049	0.517 (0.267–0.998)
MGFA classification	Univariate analysis	0.001	0.789 (0.685–0.908)
	Multivariate analysis^a^	0.007	0.811 (0.697–0.944)
Clavien-Dindo classification	Univariate analysis	0.003	0.748 (0.617–0.907)
	Multivariate analysis^a^	0.013	0.783 (0.645–0.950)
Osserman’s classification	Univariate analysis	0.006	0.680 (0.515–0.897)
QMG score	Univariate analysis	0.010	0.948 (0.910–0.987)
Preoperative crisis^b^	Univariate analysis	0.036	0.550 (0.314–0.963)
Preoperative max pyridostigmine	Univariate analysis	0.025	0.998 (0.996–1.000)
Lung function	Univariate analysis	0.005	0.738 (0.597–0.912)

We defined “status = 1” as tracheal extubation and “time” as the duration of mechanical ventilation in the analysis of Cox proportional hazard regression analyse

^a^Multivariate Cox analysis by forward step

^b^Alcohol status is defined as (1) never, (2) occasional, (3) excessive, and (4) dependence. Preoperative crisis is defined as the times of myasthenic crisis before surgery

**Fig. 1:**
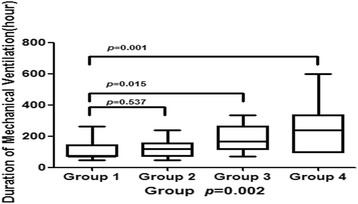
The correlation between postoperative complications and DMV, analyzed by one-way ANOVA. Sixty patients were divided into four groups based on the severity of postoperative complications as per Clavien-Dindo classification: group 1 (no complication, *n* = 18), group 2 (PLI only, *n* = 21), group 3 (PLI with other complications requiring pharmacological treatment, *n* = 14), and group 4 (PLI with other complications requiring at least invasive intervention, *n* = 7). As shown in Fig. 1, PLI, as the sole complication, did not prolong DMV compared to those without any complication (*p* = 0.537) most likely because of the use of effective antibiotics and proper respiratory management. However, PLI with other more severe complications that required at least pharmacological treatment (groups 3 and 4) seemed to remarkably prolong DMV, compared to those without any complication (*p* = 0.015 and 0.001, respectively). *p* = 0.997 (XLSTAT, Addinsoft Inc., New York, NY, USA)

Among the 70 patients, we excluded seven patients who underwent urgent thymectomy in the duration of MC to exclude confounders. As shown in Table 3, PLI occurred in 60 cases (60/63, 95.2%). Therefore, we tried to evaluate the impact of PLI on DMV and excluded three patients without PLI but with other complications. All of the PLI were grade II as per Clavien-Dindo classification. Finally, 60 patients were divided into four groups according to the severity of postoperative complications as per Clavien-Dindo classification, i.e., group 1 (no complication), group 2 (PLI only), group 3 (PLI with other complications requiring pharmacological treatment), and group 4 (PLI with other complications requiring at least invasive intervention). As shown in Fig. 1, PLI as the sole complication did not prolong DMV compared to those without any complication (*p* = 0.537); however, PLI with other more severe complications requiring at least pharmacological treatment (groups 3 and 4) seemed to remarkably prolong DMV, compared to those without any complication (*p* = 0.015 and 0.001).

**Table 3:** Postoperative complications of the 63 cases with or without PLI

Major postoperative complications	Number (%)	Clavien-Dindo classification
Total	63	
No postoperative complications	18 (28.6)	0
Incision infection	1 (1.6)	I
Postoperative lung infection (PLI) only	21 (33.3)	II
PLI + atrial fibrillation	3 (4.8)	II
PLI + sinus tachycardia	7 (11.1)	II
PLI + bacteremia	1 (1.6)	II
PLI + gastrointestinal bleeding	1 (1.6)	II
PLI + pneumothorax	3 (4.8)	II + IIIa
PLI + pleural effusion	3 (4.8)	IIIa
Hemothorax	1 (1.6)	IIIb
Mediastinal abscess	1 (1.6)	IIIb
PLI + acute cardiac failure	1 (1.6)	IVa
PLI + acute respiratory distress syndrome (ARDS)	2 (3.2)	IVa

### Risk factors of overall survival in PTMC patients

Among the 70 PTMC patients, 56 cases have valid follow-up data, and among them, four patients died of sudden deterioration of MC (three patients and one patient died within 12 and 18.8 months after the operation, respectively). We defined “status = 1” as the event of death and “time” as the survival duration. As shown in Table 4, high scores of Osserman’s classification, MGFA classification, and Clavien-Dindo classification predicted a poor prognosis in PTMC patients.

**Table 4:** Univariate analysis of risk factors that may impact overall survival

Variables	*p* value	Hazard ratio (95.0% CI for HR)
Osserman’s classification	0.017	8.723 (1.464–51.955)^a^
MGFA classification	0.007	3.562 (1.418–8.945)^b^
DMV	0.005	1.007 (1.002–1.012)^c^
Clavien-Dindo classification	0.033	1.990 (1.057–3.746)^d^
Drainage	0.010	1.002 (1.001–1.004)^e^

We defined “status = 1” as the event of death and “time” as the survival duration

Statistical powers of the Cox model were evaluated by using XLSTAT (Addinsoft Inc., New York, NY, USA) and presented as follows: (a) 1.000, (b) 1.000, (c) 0.473, (d) 0.962, and (e) 0.469

### Relationship between DMV and postoperative quality of life

We evaluated postoperative QOL in the PTMC patients as per MG-QOL15, a self-administered disease-specific questionnaire consisting of 15 items ranging from 0 to 4, i.e., from “not at all” to “very much” [16].

**Fig. 2:**
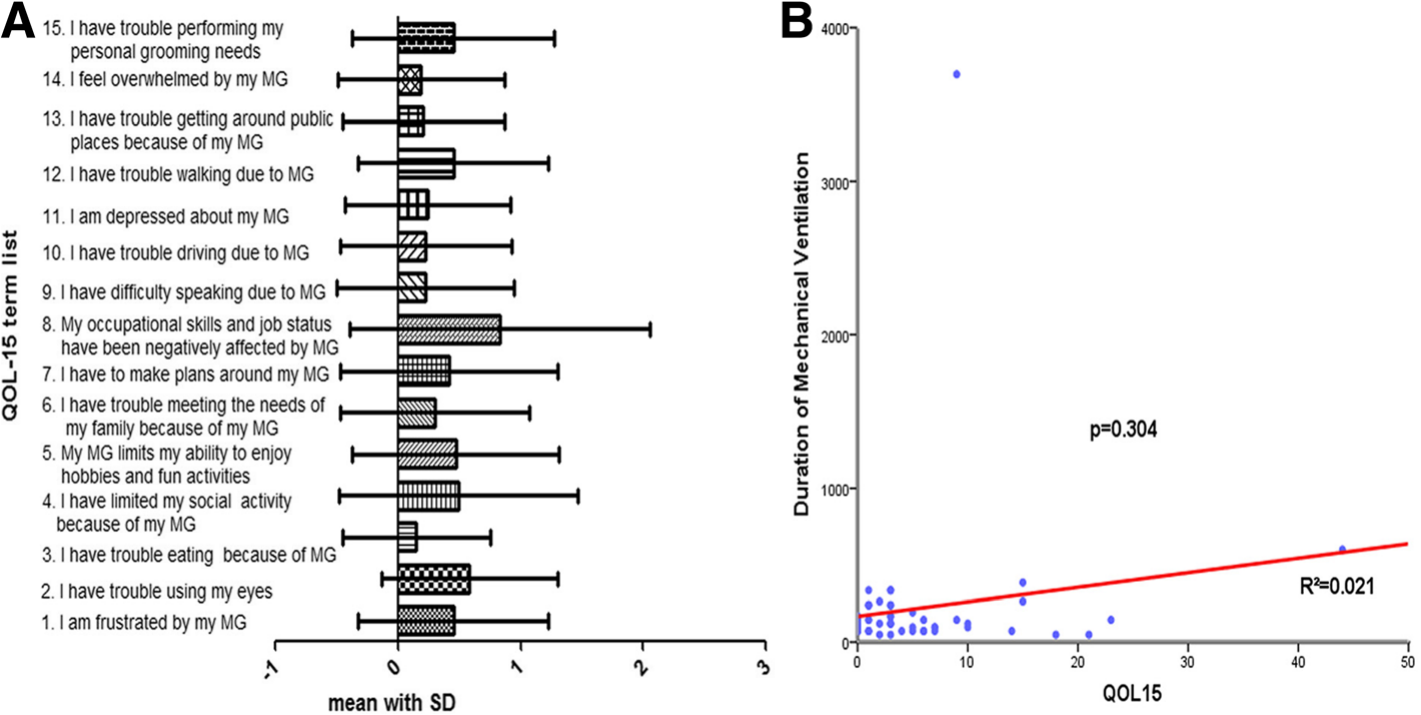
**a** The score (mean with SD) of each list in MG-QOL15, occupational skills and job status seemed to be most negatively affected by PTMC, compared to other challenges in daily life. **b** Spearman bivariate correlation analysis between DMV and QOL; DMV was not correlated with QOL (*R* ^2^ = 0.021, *p* = 0.304)

As shown in Fig. 2a, occupational skills and job status seemed to be most negatively affected in PTMC patients, compared to other challenges in daily life. As shown in Fig. 2b, the mean score of long-term (more than 1 year) postoperative QOL was 5.6, and DMV was not correlated with QOL (*R* ^2^ = 0.021, *p* = 0.304).

## Discussion

Thymectomy via video-assisted thoracoscopic surgery (VATS) or partial/total sternotomy is one of the most important therapies in the treatment of MG and has a critical role in decreasing the frequency and severity of myasthenic attacks [17]. PTMC is the primary cause of postoperative mortality and prolonged hospital stay. In our study, the morbidity of PTMC is 18.2% (70/384); this result is in accordance with other reports that range from 6 to 21.9% [18, 19].

PTMC is the most critical complication of respiratory failure resulting from serious weakness of the respiratory muscle and the accumulation of saliva and bronchial secretions in the airway [5, 20] and usually requires mechanical ventilation. However, subsequent postoperative mechanical ventilation could induce respiratory complications and increase medical costs. Therefore, early discontinuation of mechanical ventilation and extubation is very important to accelerate the rehabilitation of the cases. In our study, alcohol status, high MGFA classification, and Clavien-Dindo classification were critical independent factors that may hinder early extubation.

Interestingly, alcohol misusers were observed to have longer bleeding times during the first postoperative week, as well as a weaker surgical stress response and higher postoperative morbidity; these are most likely the result of decreased hemostatic function, immunosuppression, and subclinical cardiac insufficiency [21]. Therefore, preoperative alcohol consumption may be a more important risk factor in patients with MG than previously thought [21].

MGFA classification dissimilates to Osserman’s classification in the evaluation of severity, and it is designed to identify subgroups of patients with MG who share distinct clinical features or severity of disease that may indicate different prognoses or responses to therapy, and it is more objective and precise than Osserman’s classification [13]. In addition, the QMG score is prone to be affected by clinical classification and postintervention status and should not be used to compare severity between patients [13]. In our study, MGFA classification, rather than Osserman’s classification and QMG score, was the independent factor of DMV in PTMC, demonstrating that preoperative MGFA classification can be used to predict PTMC.

We evaluated postoperative complications by using the Clavien-Dindo classification and found that PLI, as the sole complication, did not prolong DMV, most likely because of the use of effective antibiotics and proper respiratory management. However, PLI with other more severe complications (Clavien-Dindo classification ≥2) seemed to remarkably prolong DMV. Therefore, we should focus on the treatment of these complications, e.g., atrial fibrillation, pneumothorax, and ARDS.

Lee et al. reported that after thymectomy, 2.4% (3/123) of MG patients had unchanged or worse exacerbation of symptoms or died of MG [22]. However, few studies focused on the survival of PTMC patients. In the present study, four (4/56, 7.1%) patients died of sudden deterioration of MC within 2 years after PTMC, and high scores of Osserman, MGFA, and Clavien-Dindo classifications predicted a poor prognosis in PTMC patients.

Remission and absence of generalized symptoms were favorable factors for QOL in MG patients [23], while the frequency of MG symptoms might be the primary factor that lowers QOL [24]. However, very few studies focused on long-term QOL after surgery in PTMC patients. In our study, the occupational skills and job status seemed to be remarkably negatively affected by MG and we demonstrated that DMV was not correlated with QOL.

## Conclusions

Based on the results of this study, in order to decrease the duration of mechanical ventilation, we suggest alcohol abstinence before the operation, appropriate preoperative treatment to decrease MGFA classification, and more focus on the treatment of postoperative complications. However, our study has several limitations, e.g., a limited number of the cohort from a single institution. A multi-center robust study with long-term follow-up is warranted.

## Additional file

Additional file 1: Table S1. Published information regarding the 16 variables and their potential impacts on MG. (DOC 120 kb)

## Abbreviations

AChR: Acetylcholine receptor
DMV: Duration of mechanical ventilation
MC: Myasthenic crisis
MG: Myasthenia gravis
MGFA: Myasthenia Gravis Foundation of America
OS: Overall survival
PLI: Postoperative lung infection
PTMC: Post-thymectomy myasthenic crisis
QMG: Quantitative myasthenia gravis
QOL: Quality of life
VATS: Video-assisted thoracoscopic surgery
