# Supplementary material for: Predictive factors of prolonged mechanical ventilation, overall survival, and quality of life in patients with post-thymectomy myasthenic crisis
Source: World J Surg Oncol. 2017 Aug 8;15:150. doi: 10.1186/s12957-017-1209-1 (PMC5549389; doi:10.1186/s12957-017-1209-1)
Supplement: Additional file 1: Table S1. — Published information regarding the 16 variables and their potential impacts on MG. (DOC 120 kb) [file 12957_2017_1209_MOESM1_ESM.doc]

Additional file 1: Table S1: Published information regarding the 16 variables and their potential impacts on MG.

| Variables | Published information |
| --- | --- |
| **Demographical characteristics** |  |
| Age | The prevalence and incidence of MG were higher in the older (≥50 years) group than in the younger (<50 years) group[1]. |
| BMI | The predictor of longer operation time for VATS thymectomy[2, 3], and one of the risk factors of pulmonary complications after thoracic operation[4-6] |
| Smoking Status | A close association between smoking status and symptom severity in MG[7, 8] |
| Alcohol Status | An increased postoperative morbidity among alcohol misusers[9] |
| Lung Function | A close relationship between lung function and postoperative ARDS, myasthenic crisis and mortality [10-12] |
| **Status of MG prior to thymectomy** |  |
| Preoperative crisis | Status of MG, i.e., preoperative crisis, symptom duration, Osserman Classification, MGFA Classification and QMG Score were confirmed to be the risk factors of PTMC [13-15] |
| Symptom Duration |
| Osserman’s Classification |
| MGFA Classification |
| QMG Score |
| Thymoma | More severe symptoms and poorer prognosis in the cases with Thymoma [16] |
| Maximal preoperative pyridostigmine | Thymoma patients with a large dosage of pyridostigmine bromide administration were prone to PTMC[15]. |
| **Surgical situations** |  |
| Surgical approaches | Operation situations e.g., surgical approaches, operation time and blood loss were reported to be associate with the postoperative complications, especially pulmonary infection or PTMC [16-18]. |
| Operation time |
| Blood loss |
| **Postoperative situations** |  |
| Complications classified by Clavien-Dindo Classification | Postoperative complications were confirmed to be the risk factors of PTMC [13-15] |

**References:**

1. Lee HS, Lee HS, Shin HY, Choi YC, Kim SM. The Epidemiology of Myasthenia Gravis in Korea**.** Yonsei Med J. 2016; 57**:**419-25.

2. Bachmann K, Burkhardt D, Schreiter I, Kaifi J, Schurr P, Busch C, Thayssen G, Izbicki JR, Strate T. Thymectomy is more effective than conservative treatment for myasthenia gravis regarding outcome and clinical improvement**.** Surgery. 2009; 145**:**392-8.

3. Jani-Acsadi A, Lisak RP. Myasthenic crisis: guidelines for prevention and treatment**.** J Neurol Sci. 2007; 261**:**127-33.

4. Toker A, Tanju S, Ziyade S, Kaya S, Dilege S. Learning curve in videothoracoscopic thymectomy: how many operations and in which situations? Eur J Cardiothorac Surg. 2008; 34**:**155-8.

5. Toker A, Tanju S, Sungur Z, Parman Y, Senturk M, Serdaroglu P, Dilege S, Deymeer F. Videothoracoscopic thymectomy for nonthymomatous myasthenia gravis: results of 90 patients**.** Surg Endosc. 2008; 22**:**912-6.

6. Agostini P, Cieslik H, Rathinam S, Bishay E, Kalkat MS, Rajesh PB, Steyn RS, Singh S, Naidu B. Postoperative pulmonary complications following thoracic surgery: are there any modifiable risk factors? Thorax. 2010; 65**:**815-8.

7. Gratton SM, Herro AM, Feuer WJ, Lam BL. Cigarette Smoking and Activities of Daily Living in Ocular Myasthenia Gravis**.** J Neuroophthalmol. 2016; 36**:**37-40.

8. Maniaol AH, Boldingh M, Brunborg C, Harbo HF, Tallaksen CM. Smoking and socio-economic status may affect myasthenia gravis**.** Eur J Neurol. 2013; 20**:**453-60.

9. Tonnesen H, Petersen KR, Hojgaard L, Stokholm KH, Nielsen HJ, Knigge U, Kehlet H. Postoperative morbidity among symptom-free alcohol misusers**.** Lancet. 1992; 340**:**334-7.

10. Liu LX, Hu ZJ, Zhao C. [Correlations of preoperative pulmonary function tests for esophageal cancer to postoperative acute respiratory distress syndrome]**.** Ai Zheng. 2006; 25**:**335-8.

11. Chinn S, Gislason T, Aspelund T, Gudnason V. Optimum expression of adult lung function based on all-cause mortality: results from the Reykjavik study**.** Respir Med. 2007; 101**:**601-9.

12. Choi KH, Nam TS, Lee SH, Kim MK. Preoperative pulmonary function is strongly related to myasthenic crisis after thymectomy**.** Neurol India. 2014; 62**:**164-8.

13. Zou J, Su C, Lun X, Liu W, Yang W, Zhong B, Zhu H, Lei Y, Luo H, Chen Z. Preoperative Anxiety in Patients With Myasthenia Gravis and Risk for Myasthenic Crisis After Extended Transsternal Thymectomy: A CONSORT Study**.** Medicine (Baltimore). 2016; 95**:**e2828.

14. Lu W, Yu T, Longhini F, Jiang X, Qin X, Jin X. Preoperative risk factors for prolonged postoperative ventilation following thymectomy in myasthenia gravis**.** Int J Clin Exp Med. 2015; 8**:**13990-6.

15. Wu Y, Chen Y, Liu H, Zou S. Risk factors for developing postthymectomy myasthenic crisis in Thymoma Patients**.** J Cancer Res Ther. 2015; 11 Suppl 1**:**C115-7.

16. Yu L, Zhang XJ, Ma S, Li F, Zhang YF. Thoracoscopic thymectomy for myasthenia gravis with and without thymoma: a single-center experience**.** Ann Thorac Surg. 2012; 93**:**240-4.

17. Yu S, Lin J, Fu X, Li J, Li Y, Chen B, Yang M, Zhang M, Bu B. Risk factors of myasthenic crisis after thymectomy in 178 generalized myasthenia gravis patients in a five-year follow-up study**.** Int J Neurosci. 2014; 124**:**792-8.

18. Jurado J, Javidfar J, Newmark A, Lavelle M, Bacchetta M, Gorenstein L, D'Ovidio F, Ginsburg ME, Sonett JR. Minimally invasive thymectomy and open thymectomy: outcome analysis of 263 patients**.** Ann Thorac Surg. 2012; 94**:**974-81, 981-2.
